# Life on the outside: role of biofilms in environmental persistence of Shiga-toxin producing *Escherichia coli*

**DOI:** 10.3389/fmicb.2014.00317

**Published:** 2014-07-01

**Authors:** Philippe Vogeleer, Yannick D. N. Tremblay, Akier A. Mafu, Mario Jacques, Josée Harel

**Affiliations:** ^1^Groupe de Recherche sur les Maladies Infectieuses du Porc, Département de Pathologie et Microbiologie, Faculté de Médecine Vétérinaire, Centre de Recherche d'Infectiologie Porcine et Avicole, Université de MontréalSt-Hyacinthe, QC, Canada; ^2^Food Research and Development Centre, Agriculture and Agri-Food CanadaSt-Hyacinthe, QC, Canada

**Keywords:** STEC, biofilm, sanitizers, processing plant, environment

## Abstract

*Escherichia coli* is a heterogeneous species that can be part of the normal flora of humans but also include strains of medical importance. Among pathogenic members, Shiga-toxin producing *E. coli* (STEC) are some of the more prominent pathogenic *E. coli* within the public sphere. STEC disease outbreaks are typically associated with contaminated beef, contaminated drinking water, and contaminated fresh produce. These water- and food-borne pathogens usually colonize cattle asymptomatically; cows will shed STEC in their feces and the subsequent fecal contamination of the environment and processing plants is a major concern for food and public safety. This is especially important because STEC can survive for prolonged periods of time outside its host in environments such as water, produce, and farm soil. Biofilms are hypothesized to be important for survival in the environment especially on produce, in rivers, and in processing plants. Several factors involved in biofilm formation such as curli, cellulose, poly-N-acetyl glucosamine, and colanic acid are involved in plant colonization and adherence to different surfaces often found in meat processing plants. In food processing plants, contamination of beef carcasses occurs at different stages of processing and this is often caused by the formation of STEC biofilms on the surface of several pieces of equipment associated with slaughtering and processing. Biofilms protect bacteria against several challenges, including biocides used in industrial processes. STEC biofilms are less sensitive than planktonic cells to several chemical sanitizers such as quaternary ammonium compounds, peroxyacetic acid, and chlorine compounds. Increased resistance to sanitizers by STEC growing in a biofilm is likely to be a source of contamination in the processing plant. This review focuses on the role of biofilm formation by STEC as a means of persistence outside their animal host and factors associated with biofilm formation.

## Introduction

*Escherichia coli* is a diverse species of bacterium that includes members of the normal commensal flora of humans and animals but also pathogenic strains of veterinary and medical importance. Pathogenic members are usually classified in two major groups: intestinal *E. coli* (InPEC) and extraintestinal *E. coli* (ExPEC). The latter group is typically responsible for urinary tract infections [uropathogenic *E. coli* (UPEC)], neonatal sepsis, and meningitis in humans and various infectious diseases in animals including mastitis (Kaper et al., 2004; Clements et al., 2012). InPEC are classically divided in 8 sub-groups based on the diseases they cause, their virulence factors, and phylogeny. These 8 pathotypes are: adherent-invasive *E. coli* (AIEC) associated with Crohn's disease, diffusely adherent *E. coli* (DAEC), enteroaggregative *E. coli* (EAEC), enterotoxigenic *E. coli* (ETEC), enteropathogenic *E. coli* (EPEC), Shiga-toxin producing *E. coli* (STEC) that includes enterohemorrhagic *E. coli* (EHEC), and enteroinvasive *E. coli* (including Shigella) (EIEC) (Kaper et al., 2004; Clements et al., 2012). The characteristics of each pathotype have been described in several reviews (Kaper et al., 2004; Croxen and Finlay, 2010; Clements et al., 2012).

STEC are worldwide water- and food-borne pathogens and are some of the more prominent pathogenic *E. coli* within the public sphere (Etcheverria and Padola, 2013). Cattle are an important animal reservoir of STEC and this colonization is typically asymptomatic (Ferens and Hovde, 2011). STEC can also be shed in the feces of sheep, goats, turkeys, and pigs (Heuvelink et al., 1999; Booher et al., 2002; Cornick and Helgerson, 2004; Vu-Khac and Cornick, 2008; Best et al., 2009; La Ragione et al., 2009). STEC disease outbreaks are typically associated with contaminated beef; however unpasteurized milk, contaminated drinking water, contaminated fresh produce, and unpasteurized apple cider have also been implicated (Ferens and Hovde, 2011). In addition to living within animal reservoirs, STEC can persist for prolonged periods of time in the environment, such as in water and farm soil. For example, EHEC can survive for periods greater than 8 months in water contaminated with bovine feces (Ferens and Hovde, 2011).

STEC are also a major concern in food-processing plants and contamination of beef carcasses with STEC may occur during different stages of processing such as slaughtering, dressing, chilling or cutting (Bacon et al., 2003; Koutsoumanis and Sofos, 2004). Therefore, populations of contaminating STEC are likely present on the surface of several pieces of equipment associated with slaughtering and processing. These pieces of equipment may potentially contaminate unadulterated carcasses and fresh meat products (Gill and McGinnis, 2000; Barkocy-Gallagher et al., 2001; Gill et al., 2001; Tutenel et al., 2003). The presence of STEC in beef and food processing plants has been well documented and it has been suggested that the ability to form biofilms on different surfaces is responsible for the distribution and persistence of STEC in meat processing plants (Carpentier and Cerf, 1993; Dewanti and Wong, 1995; Aslam et al., 2004; Rivera-Betancourt et al., 2004). In this review, we will explore the role of biofilm formation by STEC as a means of persistence outside their animal hosts and factors associated with biofilm formation.

## Genetic diversity of STEC

The predominant STEC serotype associated with outbreaks is O157:H7. Since it was one of the first serotypes identified as causing hemolytic uremic syndrome (HUS) and the most severe illness, EHEC O157:H7 is the most commonly reported STEC serotype in the media (Etcheverria and Padola, 2013). However, other clinically relevant serotypes have been identified and are commonly called the “the big six,” these include serotypes O26, O45, O103, O111, O121, and O145 (Wang et al., 2012). Other serotypes (e.g., O113:H21 and O91:H21) generally do not cause outbreaks but have been associated with sporadic cases of HUS (Karmali et al., 2003). Additionally, a new type of emerging STEC strain was identified after the large HUS outbreak in Germany in 2011 (Frank et al., 2011). This strain belongs to the serotype O104:H4 and combines the chromosomal backbone of a typical EAEC strain with the bacteriophage encoding Stx2 from STEC (Scheutz et al., 2011). The *stx2* gene was presumably acquired via horizontal gene transfer. This atypical Shiga-toxin producing enteroaggregative *E. coli* (STEAEC) strain will not be covered in this review because it does not fit within the classic STEC pathotype.

In addition to serotype diversity within the STEC pathotype, genetic diversity in the O157:H7 serotype is gaining ground as a source of variation in virulence between strains (Bono et al., 2007; Manning et al., 2008; Zhang et al., 2010; Shringi et al., 2012). This phenomenon is observed with different *E. coli* O157 strains, where there is a significant association between clades and the severity and duration of disease (Fukushima et al., 1999; Grant et al., 2008; Manning et al., 2008). Furthermore, geographical distribution also appears to influence the phylogeny of *E. coli* O157 populations and recent findings suggest divergent evolution of EHEC O157 in Australia and the United States (Mellor et al., 2013). Despite this diversity, most studies on STEC biofilm formation are performed with the sequenced reference strain EDL933 that was isolated from meat associated with a USA hemorrhagic colitis outbreak in 1982 (Perna et al., 2001; Manning et al., 2008). Therefore, some of the conclusions may only reflect North American strains rather than strains isolated from other continents.

## Biofilm formation by STEC

Generally, bacteria do not live freely in suspension (planktonic cells), but in complex communities called biofilms. Biofilms are aggregates of microorganisms (bacteria, fungi, algae, or protozoa) enclosed in a self-produced extracellular polymeric matrix that are attached to a biotic or abiotic surface (Costerton et al., 1999; Hall-Stoodley and Stoodley, 2009; Jacques et al., 2010). Biofilms protect bacteria from several challenges including desiccation, bacteriophages, amoebae, and biocides used in industrial processes (Costerton et al., 1999). With respect to *E. coli* biofilm formation, studies have mostly been performed with K12 strains and have been reviewed in several publications (Beloin et al., 2008; Wood, 2009). EDL933 and MG1655 share a core set of genes, including some genes involved in biofilm formation; as a result, data obtained using K12 strains are often used to infer function for STEC strains. However, such inferences are not always appropriate because there are key differences between the genomes of K12 and EDL933 including the presence of O-islands, lack of type 1 fimbriae production, and the presence of single nucleotide polymorphisms (SNP) (Perna et al., 2001; Roe et al., 2001; Welch et al., 2002; Zhang et al., 2006; Chen et al., 2013). Additionally, the expression and activity of several factors that must act at specific times and at various locations in the biofilm are required for proper biofilm formation (Beloin et al., 2008; Wood, 2009).

## Differences between STEC and K12 that may influence biofilm formation

As stated above, there are key differences between K12 and STEC strains that may have major influences on biofilm formation. For example, the EDL933 genome possesses 177 O-islands (OI), several of which encode fimbrial adhesins (Perna et al., 2001). However, the presence of a gene in a genome does not guarantee its expression. As an example, type 1 fimbriae are associated with biofilm formation in K12 strains, but a deletion in the *fim* regulatory region abolished type 1 fimbriae expression in *E. coli* O157:H7 (Roe et al., 2001; Beloin et al., 2004). Therefore, type 1 fimbriae do not play a role in biofilm formation by *E. coli* O157:H7. These data highlight the fact that findings for K12 do not always represent biological processes for all *E. coli* subtypes. Furthermore, many groups have demonstrated that STEC biofilm formation is more dependent on the strain than the serotype. This could be explained by the presence of SNP that result in premature stop codons in genes encoding adhesins or RpoS, the stationary phase sigma factor that is important for biofilm formation and regulation (Zhang et al., 2006).

In addition to differences at the genomic level, there are key differences in the transcript profiles of K12 and EHEC strains for biological processes involved in the interactions with lettuce leaves (Fink et al., 2012) including genes that may be involved in biofilm formation. Differences in the transcriptomes of K12 and STEC strains could be explained by the presence of additional regulators encoded within genomic islands and changes in promoter regions. For example, the genomic island OI-47 of *E. coli* O157:H7 contains a gene, *vmpA*, coding for a c-di-GMP phosphodiesterase that is specific for EHEC O157:H7 and VmpA was shown to influence the regulation of biofilm formation (Branchu et al., 2013). Furthermore, recent findings have highlighted differences in EHEC and EPEC promoter regions that result in the differential regulation of an outer-membrane protease (Thomassin et al., 2012). Taken together, these differences indicate that biofilm data obtained with K12 strains or other pathotypes are not always directly relevant to STEC strains. Therefore, it is important that biofilm formation be studied in STEC.

## Steps in biofilm formation

**Figure 1:**
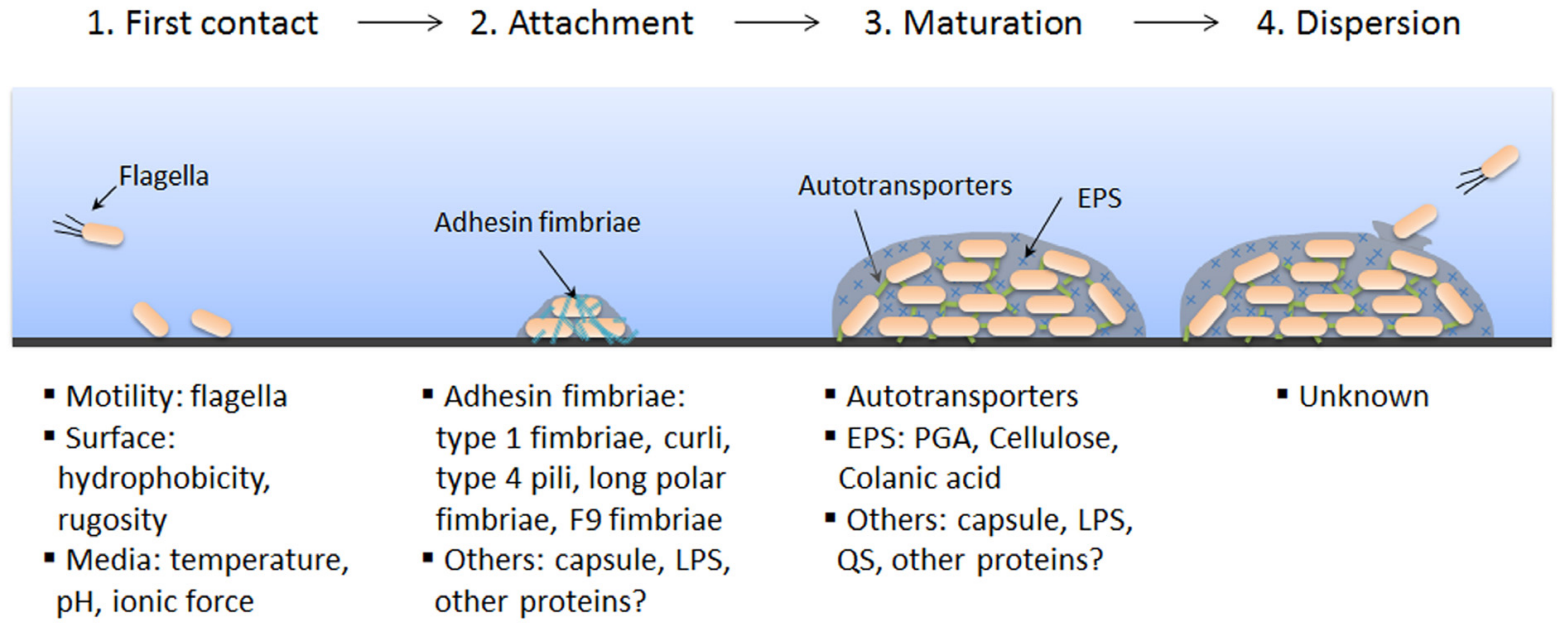
**Schematic representation of biofilm formation and STEC factors associated with each step**. Biofilm formation is a dynamic and complex process influenced by several bacterial and/or environmental factors. Biofilm formation occurs in four steps: first contact, attachment, maturation, and dispersion. Factors that are known to play a role in STEC biofilm formation are listed below the corresponding step. It should be noted that biofilm formation in STEC is strain dependent and factors presented in this figure are not necessarily representative of all STEC biofilms. PGA, poly-N-acetyl glucosamine; EPS, extracellular polymeric substances; LPS, lipopolysaccharides; QS, quorum sensing.

Biofilm formation requires specific steps and is typically described as a four-step process: initial contact, attachment, maturation, and dispersion (Figure 1).

### Initial contact

The first step in biofilm formation is reversible attachment to a surface; this is dependent on a balance of attractive and repulsive forces between the bacteria and the surface. Both environmental and bacterial factors are important for this interaction. Attachment is influenced by environmental conditions such as temperature, pH, ionic force of the medium, and the rugosity of the surface in addition to bacterial properties such as hydrophobicity and motility (Fletcher, 1988; Pratt and Kolter, 1998; Danese et al., 2000). Furthermore, flagella-driven motility is considered to be an important factor during the initial step of biofilm formation by *E. coli* because K12 strains lacking flagella do not produce biofilms. Additionally, Chen and collaborators recently suggested that flagella-driven motility is also involved in biofilm formation of non-O157:H7 STEC (Chen et al., 2013).

### Attachment

The second step in biofilm formation is irreversible attachment, which is often influenced by the presence of surface structures such as fimbrial adhesins (Beloin et al., 2004). Many classes of fimbriae have been implicated in the attachment of STEC to surfaces, including type 1 fimbriae, curli, type 4 pili, long polar fimbriae, and F9 fimbriae (Farfan and Torres, 2012).

### Maturation

The third step in biofilm formation is maturation. During biofilm maturation, bacteria continue to multiply and produce extracellular matrix. At this stage, the biofilm adopts a three-dimensional structure. This growth is mostly due to bacterium–bacterium interactions; several surface proteins and extracellular matrix components are involved in bacterial adhesion and biofilm architecture (Beloin et al., 2008). Two important factors for this step have been identified in *E. coli*: autotransporters for cell-cell interactions and exopolysaccharides (EPS) for the matrix architecture (Beloin et al., 2008).

#### Important factors for biofilm maturation: autotransporters

Autotransporter adhesins, which are members of the type V secretion system, have been associated with autoaggregation and biofilm formation. Screens in STEC strains have identified 9 autotransporter genes: chromosome encoded *agn43, cah, ehaA, ehaB, ehaD, ehaG, saa*, and *sab* and plasmid-encoded *espP* (Torres et al., 2002; Wells et al., 2008, 2009; Herold et al., 2009; Puttamreddy et al., 2010). The protein products of each of these genes have been associated with biofilm formation (Torres et al., 2002; Wells et al., 2008, 2009; Herold et al., 2009; Puttamreddy et al., 2010). A comparative study of three autotransporter genes (*agn43*, *cah*, and *ehaA*) among 51 STEC strains found that the presence of autotransporter genes within the genome was variable among STEC serotypes (Biscola et al., 2011). Specifically, *agn43* was present at a higher frequency in non-O157 strains than O157 strains while the frequency of *cah* is higher in O157 strains compared to non-O157 strains (Biscola et al., 2011).

In addition to the autotransporter *espP*, the pO157 plasmid encodes the enterohemolysin translocator *ehxD*, whose protein product was identified as a mediator of biofilm formation, indicating that pO157 is essential for biofilm formation (Puttamreddy et al., 2010). Large plasmids similar to pO157, encoding *espP*, and *ehxD* can also be found in many non-O157 EHEC strains (Brunder et al., 1999; Caprioli et al., 2005; Verstraete et al., 2013).

#### Important factors for biofilm maturation: EPS

The *E. coli* biofilm matrix can be composed of three different EPSs: poly-N-acetyl glucosamine (PGA), colanic acid, and/or cellulose. The genes encoding proteins that are involved in the synthesis of these polysaccharides are present in the genomes of STEC strains EDL933 and Sakai (Hayashi et al., 2001; Perna et al., 2001), however, their role in biofilm formation has not been directly established in these strains. However, O157:H7 mutants lacking genes encoding proteins needed to make PGA, cellulose, or colanic acid were unable to adhere to alfalfa sprouts (Matthysse et al., 2008). Furthermore, cellulose production was correlated with biofilm formation in O157 strains (Biscola et al., 2011; Lee et al., 2011). Cellulose production is, however, variable and dependent on both the bacterial strain and environmental conditions (Beloin et al., 2008). Colanic acid is produced by *E. coli* O157:H7, but there is limited data for other STEC serotypes (Beloin et al., 2008). The production of colanic acid protects *E. coli* O157:H7 against osmotic and oxidative stress, suggesting that colanic acid may be implicated in STEC biofilm formation, however, this remains to be tested directly (Yeh and Chen, 2004).

#### Important factors for biofilm maturation: other factors

Lipopolysaccharides (LPS) and capsules, which are surface structures, have been implicated in biofilm formation by *E. coli*. Mutations affecting LPS synthesis affect the ability of *E. coli* K12 strains to adhere to surfaces and form biofilms (Genevaux et al., 1999; Beloin et al., 2008). Similar observations were noted for *E. coli* O157:H7 strain EDL933, where O-antigen transposon mutants could not form biofilms (Puttamreddy et al., 2010).

Capsules are known to mask bacterial surface adhesins and often have an indirect effect on biofilm formation (Schembri et al., 2004). According to Whitfield, capsule polysaccharides produced by some EHEC strains belong to the *E. coli* group 4 capsule, which is composed of the same sugar repeats as the LPS O-antigen and acetamido sugars in their repeat-unit structures (Whitfield, 2006). The impact of this capsule-type on biofilm formation by STEC has yet to be investigated. If the group 4 capsule has an impact on biofilm formation, its effect might be serotype specific given the diversity of O-antigen structures among STEC. Furthermore, the capsule is only expressed and present under specific laboratory conditions in EDL933 (Shifrin et al., 2008; Thomassin et al., 2013). Therefore, the role of the capsule in *in vivo* biofilm formation might be difficult to evaluate in an *in vitro* setting given that biofilm formation is also highly dependent on growth conditions.

Curli fimbriae are structures that aggregate on the surface of cells, they promote adhesion of *E. coli* to different human cells and biofilm formation on abiotic surfaces (Olsen et al., 1989; Ben Nasr et al., 1996; Vidal et al., 1998; Cookson et al., 2002; Uhlich et al., 2006). Curli expression in some STEC strains has been associated with biofilm formation on polystyrene and stainless steel surfaces (Cookson et al., 2002; Ryu et al., 2004b; Uhlich et al., 2006). However, curli expression, which is strain dependent and serotype independent, is not essential for biofilm formation (Wang et al., 2012). Additionally, curli can interact with cellulose to create networks resulting in the formation of a hydrophobic extracellular matrix (Zogaj et al., 2001; Gualdi et al., 2008). Curli are thought to facilitate initial cell–surface interactions and, subsequent cell–cell interactions (Cookson et al., 2002; Uhlich et al., 2006). Curli are encoded in two divergently transcribed operons: the *csgBA* operon encodes the structural components and the *csgDEFG* operon encodes the regulator (CsgD) and the export machinery (CsgE-G) (Hammar et al., 1995). Both operons are found in the EHEC O157:H7 EDL933 and Sakai reference strains (Hayashi et al., 2001; Perna et al., 2001). Curli production is tightly controlled and complex; several transcriptional regulators (EnvZ/OmpR, CpxR, RcsCDB, RpoS, H-NS, IHF, Crl, and MlrA) and conditions (temperature, osmolarity, pH, and oxygenation) control curli expression, which involves a network of interactions (Dorel et al., 1999; Prigent-Combaret et al., 2001; Brombacher et al., 2003; Gerstel et al., 2003; Jubelin et al., 2005; Vianney et al., 2005). The complex regulatory network of curli expression is thought to be fine-tuned to allow for the colonization of specific niches by *E. coli* (Prigent-Combaret et al., 2001; Kikuchi et al., 2005).

#### Important factors for biofilm maturation: quorum sensing

During the different steps of biofilm formation the bacterial cell population density fluctuates and gene expression varies. To coordinate gene expression, bacteria communicate using quorum sensing (QS) systems (Walters and Sperandio, 2006). QS systems are based on the secretion and/or recognition of signal molecules called autoinducers (AIs). Three types of AIs have been identified: AI-1, AI-2, and AI-3. Both AI-2 and AI-3 are produced, secreted, and recognized by *E. coli* strains including STEC (Walters and Sperandio, 2006). *E. coli* strains do not produce AI-1; however their genome encodes *sdiA*, the AI-1 sensor, which is a *luxR* homolog. This enables *E. coli*, including STEC strains, to recognize acyl-homoserine lactone (AHL), the signal molecule for AI-1, secreted by others bacterial species. In Sharma et al. (2010) demonstrated that SdiA acts as a repressor of curli and flagellar gene expression. An O157:H7 Δ*sdiA* strain had increased curli fimbriae and biofilm production, suggesting that the AI-1 system has a negative impact on biofilm formation (Sharma et al., 2010). LuxS, a metabolic enzyme also found in STEC strains, is primarily involved in the conversion of ribosyl-homocysteine into homocysteine and 4,5-dihydroxy-2,3-pentanedione, which is the precursor for AI-2 (Schauder et al., 2001). Biofilm formation was enhanced when AI-2-like molecules were added to an O157:H7 *luxS* deletion strain (Lu et al., 2005; Bansal et al., 2008; Vikram et al., 2010). Furthermore, AI-3 and host-produced epinephrine/norepinephrine are recognized by the QseBC two component system (Walters and Sperandio, 2006). The addition of epinephrine and norepinephrine increases EHEC motility and biofilm formation, while the addition of indole attenuates these phenotypes (Bansal et al., 2007). Moreover, motility and biofilm formation by a *qseC* deletion strain were reduced by half when compared to the wild type strain (Yang et al., 2014).

### Dispersion

The final step in biofilm formation is the detachment of bacteria from the biofilm and their dispersal, which contributes to the transmission of bacteria. Dispersal is a complex process that involves several environmental signals and effectors and no single dispersal mechanism is used by all bacterial species. As described above, bacteria generally switch from a planktonic to a biofilm lifestyle by sensing environmental changes. Biofilm dispersal has recently been reviewed in detail (Kaplan, 2010). Dispersal is the least understood step in biofilm formation for all bacterial species and has not been investigated for STEC (Figure 1). In *E. coli* other than STEC, modulation of crucial surface structures, such as type IV bundle-forming pili (BFP) in EPEC and aggregative adherence fimbriae (AAFs) in EAEC, results in the detachment of bacteria from the biofilm and surface (Knutton et al., 1999; Sheikh et al., 2002; Velarde et al., 2007). For example in EAEC, positively charged AAFs extend away from the surface of the bacterial cell to mediate surface-adherence when dispersin is produced, because dispersin binds to and neutralizes LPS charge (Sheikh et al., 2002; Velarde et al., 2007). When dispersin is down-regulated, the positively charged AAFs collapse on the bacterial surface due to their interaction with negatively charged LPS. As a consequence of this collapse, AAFs no longer adhere to surfaces and the biofilm disperses (Sheikh et al., 2002; Velarde et al., 2007). Mechanisms involved in biofilm detachment are of increased interest, because the understanding of these mechanisms could lead to the development of clinical or industrial tools to remove biofilms.

## Survival in the environment: is it biofilm mediated?

**Figure 2:**
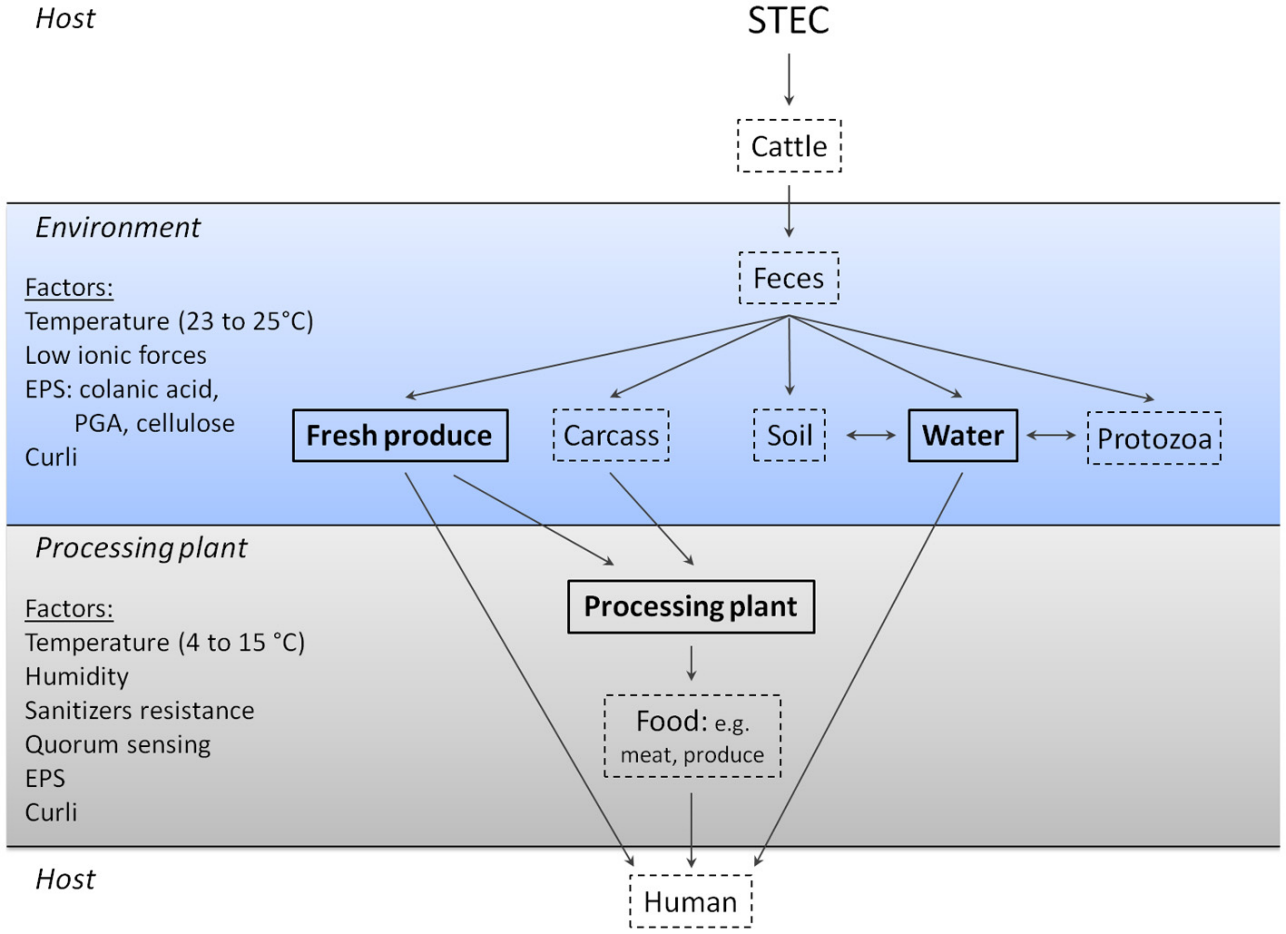
**Role of biofilm formation in the transmission and persistence of STEC outside of an animal host**. Several studies demonstrated that STEC can persist as a biofilm on fresh produce, in water, and in processing plants. In some cases, factors contributing to biofilm formation have been identified. Texts in bold and framed by solid lines indicate environments where STEC biofilms are identified and participate in the persistence of STEC; dashed lines indicate environments where STEC biofilms are hypothesized to be present. It is currently unknown if STEC biofilm formation plays a role in the colonization of cattle and humans or in STEC survival in feces, soil, protozoans, carcasses, and processed food. PGA, poly-N-acetyl glucosamine; EPS, extracellular polymeric substances; LPS, lipopolysaccharides; QS, quorum sensing.

STEC contamination of the environment and food-processing plant can occur several different ways (Figure 2). STEC are typically shed in the feces of cattle and this will contaminate the hide and farm environment (Elder et al., 2000; Aslam et al., 2003). STEC that are present in feces can contaminate manure and, consequently, soils either through manure runoff or manure applied to fields (Gagliardi and Karns, 2000; Solomon et al., 2002; Van Elsas et al., 2011). At this stage, STEC may persist and grow on fresh produce such as lettuce and can be internalized and survive within plant tissue via a mechanism that is not fully understood (Seo and Frank, 1999; Jeter and Matthysse, 2005; Tyler and Triplett, 2008). Furthermore, manure applied to fields often ends up in ground or surface water through runoff; this water is often used to irrigate fields and water crops (Ribeiro et al., 2012). As a consequence, fields and crops that were not treated with manure can become contaminated with STEC. All of these contribute to the contamination and spread of STEC in the environment. STEC can survive in soil, on fresh produce, manure, and river water, which is hypothesized to be associated with the ability of STEC to form biofilms.

### Soil and manure

Survival of *E. coli* O157:H7 in soil and manure is greatly influenced by microbial diversity; EHEC survival is at its highest when diversity is low (Vidovic et al., 2007; Van Overbeek et al., 2010; Ibekwe et al., 2011; Van Elsas et al., 2011). On one hand, survival of STEC within soil and manure is also hypothesized to be associated with the absence of various protozoa that graze on STEC (Ravva et al., 2010). On the other hand certain protozoa are proposed to act as a transmission vehicle for EHEC (Chekabab et al., 2012). Taken together these results strongly suggest that STEC survival is influenced by the environmental microcosm. There is, however, little evidence to indicate that STEC are able to form or integrate into biofilms within manure, soil, or in the farm environment. Therefore, there is a need for studies that investigate the role of biofilms in promoting STEC survival within these environments.

### Water

Biofilms containing STEC have been detected in freshwater streams that drain or are connected to agricultural land (Cooper et al., 2007; Maal-Bared et al., 2013). It is unknown if STEC can act as the pioneer bacteria in environmental biofilms, but this possibility is unlikely because periphytic *E. coli* isolates appear to form biofilms more readily than human and/or bovine isolates (Moreira et al., 2012). The presence of STEC in environmental biofilms may be explained by the finding that biofilm negative *E. coli* O157:H7 strains are able to integrate into pre-established biofilms formed by other *E. coli* strains (Uhlich et al., 2010). Therefore, it is likely that STEC can integrate into pre-existing biofilms.

Mixed-species biofilms formed in rivers and river sediments are also of particular interest because they provide ample opportunity for genetic exchange between bacteria (Maal-Bared et al., 2013). Furthermore, the environment within the biofilm is suitable for the transduction of *stx*-encoding phages carried by STEC (Solheim et al., 2013). The transfer of these phages is responsible for the spread of *stx* genes among *E. coli* species. Such genetic exchanges could contribute to the emergence of new pathogenic *E. coli* and give rise to the next outbreak strain.

### Fresh produce

Several genes and/or structures associated with biofilm formation have been identified as important factors for plant colonization by STEC. For example, PGA, cellulose, and colanic acid play a role in *E. coli* O157 binding to sprouts and tomato root segments (Matthysse et al., 2008). Furthermore, PGA is essential for binding to sprouts and cellulose and colanic acid increase the efficiency of this attachment (Matthysse et al., 2008). These findings provide evidence that the biofilm matrix associated polysaccharides are crucial for attachment to plants. In addition, these EPSs are expressed under environmental conditions [i.e., room temperature (23–25°C)], in low-ionic-strength medium, and during nutrient limitation by several *E. coli* strains (Gottesman and Stout, 1991; Matthysse et al., 2008). The expression of matrix polysaccharides in environments similar to those encountered in the presence of fresh produce further supports the likelihood that biofilm formation plays a role in STEC survival on produce.

Curli fimbriae improve the adherence of *E. coli* O157:H7 to spinach leaves, and interestingly, the improved adherence was found to be independent of cellulose production (Macarisin et al., 2012). Biofilm modulation genes (*ycfR* and *ybiM*) are also significantly up-regulated when *E. coli* O157:H7 interacts with lettuce roots (Hou et al., 2013). Furthermore, a Δ*ycfR* strain was unable to attach to or colonize lettuce roots (Hou et al., 2013). Taken together these studies suggest that certain factors involved in biofilm formation improve the environmental fitness of STEC, especially in the context of plant colonization.

### Biofilms and protozoa

Protozoans living in soil, manure, and rivers probably prey on STEC living in the environment (Ravva et al., 2010). Recent studies have shown that EDL933 can survive in *Acanthamoeba castellanii* and replicate within *Acanthamoeba polyphaga*, protozoa commonly found in soil, water, and fecal slurry (Barker et al., 1999; Chekabab et al., 2012). It was suggested that such protozoa could also serve as a transmission vehicle for EHEC (Chekabab et al., 2012). In support, the presence of the Stx-encoding prophage increases the survival of STEC isolates in the presence of *Tetrahymena pyriformis* or *Tetrahymena thermophila* (Steinberg and Levin, 2007; Mauro et al., 2013). Protozoa are known to graze on biofilms and ciliates and flagellates differently influence biofilm communities (Wey et al., 2012). For example, *Glaucoma* and *Tetrahymena* species (ciliates), expel vesicles containing viable *E. coli* O157:H7, whereas *Colpoda steinii* and *Acanthamoeba palestinensis* (flagellates) do not (Gourabathini et al., 2008). Furthermore, protozoans graze less on biofilm communities than on their planktonic cells, suggesting that biofilms may offer some protection from protozoan predation (Wey et al., 2012).

## STEC biofilms in processing plants, a potential source of meat and produce contamination

In addition to forming biofilms under environmental conditions and on plants, STEC are able to form biofilms on different surfaces often found in meat processing plants, such as stainless steel, polystyrene, glass, polyurethane, and high-density polyethylene (Dewanti and Wong, 1995; Dourou et al., 2011; Nesse et al., 2013). The introduction of contaminated food into processing plants results in the spread of STEC and contamination. As an example, it was estimated that the prevalence of *E. coli* O157 on cattle entering the slaughter floor may range from 10 to > 70% (Woerner et al., 2006). In the meat industry, contamination of surfaces with STEC can be traced to the entry of contaminated hides. Fecal contamination of hides occurs both directly and indirectly during cattle production and transit. Currently, disinfection protocols are used to try to limit the entry of STEC into slaughterhouses and processing plants. Despite the common use of disinfection protocols, STEC contamination of food still occurs, which according to the Center for Disease Control and Prevention (CDC) and Public Health Agency of Canada (PHAC) shows that disinfections protocols do not always prevent contamination (http://www.cdc.gov/ecoli/ and http://www.phac-aspc.gc.ca/fs-sa/fs-fi/ecoli-eng.php). The persistence of STEC in the presence of disinfectants gives rise to the probability that STEC survive and grow within a biofilm in processing plants (Stopforth et al., 2003; Ryu et al., 2004a; Uhlich et al., 2006; Fouladkhah et al., 2013). In this section we will focus on STEC biofilm formation and associated factors in processing plants.

In the processing plant environment, temperatures are normally controlled and maintained between 4 and 15°C. Many studies have shown that STEC are able to grow in a biofilm within this temperature range (Dourou et al., 2011; Fouladkhah et al., 2013; Nesse et al., 2013). For example, *E. coli* O157:H7 is able to colonize surfaces in contact with beef at 15°C (non-production hours temperature) and 4°C (production hours temperature) (Dourou et al., 2011; Fouladkhah et al., 2013). Interestingly, *E. coli* O157:H7 attachment increased at 4°C over time in the presence of a fat-lean tissue homogenate (Dourou et al., 2011). Furthermore, *E. coli* O157:H7 EDL933 is able to adhere and produce a dense biofilm on surfaces that are not favorable for its attachment when collagen I is present, which is a muscle fibrous extracellular matrix protein (Chagnot et al., 2013). In addition to form biofilms in meat homogenates, *E. coli* O157:H7 is also able to form biofilms on stainless steel when grown in spinach leaf lysates (Carter et al., 2012). Environmental conditions such as temperature and culture broths containing meat or vegetable residues can affect the expression of genes controlled by QS. For example, it was shown that *E. coli* O157:H7 biofilms produce large amounts of AI-2 when cultured in pork, beef or spinach broth (Silagyi et al., 2009). Based on this evidence, it is possible that QS drives biofilm formation in meat processing plants.

Fouladkhah et al. showed that the use of quaternary ammonium compound-based and peroxyacetic-based chemical sanitizers on biofilms that had matured for 1 week were more effective at 4°C than 25°C. However, these commercial sanitizers used at concentrations recommended to kill planktonic STEC were not able to kill or remove STEC biofilms from stainless steel surfaces (Fouladkhah et al., 2013). Furthermore, curli fimbriae, due to their amyloid properties, can protect bacteria from antibacterial agents like chlorine or quaternary ammonium sanitizers (Uhlich et al., 2006; Wang et al., 2012). It has been shown that tolerance of sanitizers by STEC in biofilms do not depends on serotype but on strain (Wang et al., 2012). It was also shown that at 100% relative humidity (RH), *E. coli* O157:H7 biofilms were more resistant to sanitizers than at lower RH (Bae et al., 2012). Furthermore, large biofilms were more resistant to cleaning and disinfection protocols and repeated treatment could results in the presence of viable but non-culturable *E. coli* O157:H7 that were able to regrow as a biofilm on polyurethane (Marouani-Gadri et al., 2010). Taken together, these data indicate that sanitizer efficacy may be limited against STEC growing within a biofilm community.

Interestingly, non-pathogenic bacteria isolated from processing plants, such as *Comamonas testosterone, Acinetobacter calcoaceticus, Burkholderia caryphylli*, and *Ralstonia insidiosa*, can initiate biofilm formation and may allow *E. coli* O157:H7 to integrate within a pre-formed biofilm, resulting in a mixed biofilm (Marouani-Gadri et al., 2009a; Habimana et al., 2010; Liu et al., 2014). For example, *C. testosteroni* can enhance the ability of *E. coli* O157:H7 to form biofilms (Marouani-Gadri et al., 2009a). The presence of *C. testosteroni* within the biofilm did, however, decrease the number of colony forming units of *E. coli* O157:H7 following chemical treatment when compared to chemical treatment of a single species *E. coli* O157:H7 biofilm (Marouani-Gadri et al., 2010). These data suggest that the presence of non-pathogenic bacterial species has a large influence on the ability of STEC to persist within the processing plant; due to the potential impact of these data, these findings merit further investigation.

The ability to secrete EPS is related to biofilm formation on stainless steel surfaces, but it was shown that overproduction of the EPS inhibits the initial attachment of *E. coli* O157:H7 (Ryu et al., 2004a). EPS production may also protect *E. coli* O157:H7 from sanitizer treatments (Ryu et al., 2004a; Ryu and Beuchat, 2005). As with curli, EPS production may not be essential for biofilm formation on stainless steel by bacterial pathogens, including STEC. In addition, it has been shown that bacteria producing little or no EPS, including *E. coli* O157:H7 could colonize a mature biofilm formed by EPS-producing bacteria (Castonguay et al., 2006; Klayman et al., 2009; Dourou et al., 2011). Although sanitizers are able to reduce or totally kill STEC within biofilms, it is possible that recolonization by STEC or other bacteria will be easier if cleaning protocols do not completely remove the biofilm matrix.

In addition to the protection offered by the biofilm matrix against sanitizers, it is well established that for *E. coli* in general, a slow-growing and dormant subpopulations are highly tolerant to antibacterial treatments (Lewis, 2010). Cells from this subpopulation are called multidrug tolerant persister cells and are dormant variants that emerged from regular cells (Lewis, 2010). The emergence of persister cells occurs at a higher frequency within biofilm populations than planktonic populations (Lewis, 2010). This non-heritable variation could permit STEC to survive the sanitation process and these individual cells could remain encased in the biofilm matrix. These cells could then contribute to the reestablishment of a STEC biofilm or population within the processing plant.

## Can STEC biofilms be removed?

It is known that STEC biofilms are more resistant to sanitizers than their planktonic counterparts (Wang et al., 2012). In recent years, many studies have focused on cleaning and disinfection procedures using physical and chemical methods. Three primary chemical compounds are used as sanitizers in the food service industry: chlorine-based cleaners, quaternary ammonium, and iodine sanitizers. Because of the toxicity of sanitizer residues and/or increased bacterial resistance to these decontamination reagents (Stopforth et al., 2003; Houari and Di Martino, 2007; Marouani-Gadri et al., 2009b; Hou et al., 2010; Wang et al., 2012), alternative molecules that are preferentially natural with low human and animal toxicity are being tested for their effect on biofilms.

Many essential oils have been shown to have good antibiofilm activity against food-borne pathogens (Giaouris et al., 2013). Perez-Conesa et al. have shown that surfactant micelles loaded with eugenol or carvacrol, two essential oils isolated from clove and thyme, are able to kill *E. coli* O157:H7 inside a biofilm. However, the biofilm matrix remains attached to the surface (Perez-Conesa et al., 2006, 2011), making reformation of a biofilm a dangerous possibility. While essential oils target cell viability, the best way to remove and prevent reformation of a biofilm on a surface is to degrade the EPS surrounding the bacteria by enzymatic treatment (Gibson et al., 1999; Lequette et al., 2010). A combination of an antimicrobial agent to kill cells within the biofilm with a food-grade agent able to remove the entire biofilm matrix could be a solution to reduce and potentially remove *E. coli* O157:H7 biofilms from processing plants. Others strategies such as bacteriophage treatments of *E. coli* O157:H7 biofilms have also been investigated. The KH1 bacteriophage reduces the population of O157:H7 cells attached to stainless steel, but not those incased within a biofilm matrix (Sharma et al., 2005). The effect of combined techniques such as steam and lactic acid (Ban et al., 2012), aerosolized sanitizers (Park et al., 2012), UV and dry heat (Bae and Lee, 2012) were also studied and have the potential to control STEC O157:H7 biofilms found on surfaces present in the food industry. The best approach for controlling STEC biofilm should kill *E. coli* O157:H7 within the biofilm and remove the biofilm matrix from the contaminated surface. For example, a combination of steam and lactic acid were able to kill *E. coli* O157:H7 and remove the biofilm matrix from stainless steel surfaces (Ban et al., 2012). Further studies should investigate the effect of antibiofilm molecules on the dispersal of biofilms and also focus on mixed biofilms containing both non-pathogenic and STEC bacteria.

## Conclusion

Contamination of the environment and processing plants with cow feces containing STEC is a major concern for food and public safety, especially since STEC can survive for prolonged periods of time outside its host. Biofilm formation appears to contribute significantly to STEC survival on produce, in rivers, and in processing plants. Several factors involved in biofilm formation such as curli, cellulose, PGA, and colanic acid are involved in plant colonization and attachment to different surfaces often found in meat processing plants. However, the factors involved in STEC survival within biofilms in rivers remain unknown. Furthermore, STEC biofilm formation on farms, in manure, and in soil has not been thoroughly explored despite the presence and persistence of STEC in these environments. The Stx toxin, which is a key factor in human host pathology, also appears to be an important factor for STEC survival against protozoan predation. In the food industry, resistance to sanitizers improves the ability of STEC to persist in the processing plant. Despite the development of new strategies to eradicate biofilms formed by food-borne pathogens, no effective solutions to remove STEC biofilms from surfaces have been identified. Therefore, future research should focus on the identification of factors promoting STEC survival, especially non-O157 STEC, and the persistence of STEC in environmental biofilms on the farm.

### Conflict of interest statement

The authors declare that the research was conducted in the absence of any commercial or financial relationships that could be construed as a potential conflict of interest.
